# A contextual integrity approach to genomic information: what bioethics can learn from big data ethics

**DOI:** 10.1007/s11019-024-10211-0

**Published:** 2024-06-12

**Authors:** Nina F. de Groot

**Affiliations:** Department of Philosophy, Faculty of Humanities, VU University Amsterdam, Amsterdam, the Netherlands

**Keywords:** Ethics, Genetic data, Genomics, Contextual integrity, Informed consent, Privacy

## Abstract

Genomic data is generated, processed and analysed at an increasingly rapid pace. This data is not limited to the medical context, but plays an important role in other contexts in society, such as commercial DNA testing, the forensic setting, archaeological research, and genetic surveillance. Genomic information also *crosses* the borders of these domains, e.g. forensic use of medical genetic information, insurance use of medical genomic information, or research use of commercial genomic data. This paper (1) argues that an informed consent approach for genomic information has limitations in many societal contexts, and (2) seeks to broaden the bioethical debate on genomic information by suggesting an approach that is applicable across multiple societal contexts. I argue that the contextual integrity framework, a theory rooted in information technology and big data ethics, is an effective tool to explore ethical challenges that arise from genomic information within a variety of different contexts. Rather than focusing on *individual control* over information, the contextual integrity approach holds that information should be shared and protected according to the norms that govern certain distinct social contexts. Several advantages of this contextual integrity approach will be discussed. The paper concludes that the contextual integrity framework helps to articulate and address a broad spectrum of ethical, social, and political factors in a variety of different societal contexts, while giving consideration to the interests of individuals, groups, and society at large.

## Introduction

Genomic data applications are not limited to the medical (research) context, but play an increasingly important role in other contexts in society, such as the commercial setting, the forensic setting, archaeological research, and genetic surveillance. This is reinforced by modern technologies that can generate, process, and analyse genomic data at an increasingly rapid pace. Genomic data applications are expanding, such as those in direct-to-consumer genomic testing, and the COVID-19 pandemic has accelerated the use of genomic data (Wan et al. 2022). It also becomes easier to share genomic data *across* different contexts, such as forensic use of medical genetic data (De Groot, Van Beers, Decock, et al., 2021), commercial drug development use of recreational genomic testing data (Van Dijck and Poell 2016), insurance use of medical genetic data (Pugh 2021) and forensic use of commercial genetic data (De Groot et al. 2021). The rapid growth of genomic datasets and the sharing of these datasets raise significant challenges, such as those revolving around privacy and effective governance (Bonomi et al. 2020). When genomic information is employed beyond the medical setting, we need bioethical reflection that *equally* looks beyond that setting. We need to look further than approaches based on individual informed consent and individual control, because although these are often useful for the medical setting, they have significant limitations in other societal contexts when it comes to genomic information.

To confront this challenge, this paper aims (1) to show why an informed consent approach for genomic information has limitations in many societal contexts, and (2) to broaden the bioethical debate on genomic information by presenting an approach that is applicable across multiple societal contexts. I argue that the contextual integrity framework (Nissenbaum 2009), a theory rooted in information technology and big data ethics, is a helpful approach to ethical reflection on genomic information. More precisely, it is an effective tool to explore ethical challenges that arise from genomic information within a variety of contexts. The contextual integrity framework has been developed as a response to privacy concerns regarding contemporary information technology systems in which complete individual control of information seems no longer to be feasible. Rather than focusing on *individual control* over information, the contextual integrity approach holds that information should be shared and protected according to the norms that govern certain distinct social contexts, such as the healthcare context or the workplace context.

There is a need to look further than individual control, individual privacy, and individual informed consent within genomics and genetics, because genetic information is often *shared* with other people. Many authors have called for approaches to genetics that move beyond the focus on the individual (Knoppers and Beauvais 2021; Parker and Lucassen 2004; Prainsack and Buyx 2013; Widdows 2013). However, these debates predominantly focus on the clinical or research setting. Genomics can benefit from approaches in the information technology domain, as this field already has a lot of experience with adapting to rapidly evolving technologies that come with enormous amounts of data that cross different contexts and with addressing the limitations of individual-focused concepts, such as individual privacy, autonomy, and consent.

This paper is structured as follows. First, I discuss the limitations of an individual-based bioethical approach to genomic information flows. Next, I discuss Nissenbaum’s framework of contextual integrity. Subsequently, I argue that the contextual integrity approach is well-equipped to face the challenges of genomics. It contributes to moving the bioethical debate on genomic information beyond the individual and beyond the medical context. Also, it enables consideration for *group* interests. I discuss these advantages of the contextual integrity framework for genomic information by exploring multiple cases across several social contexts (including research, clinical, forensic, and commercial contexts). I conclude that the contextual integrity approach is a helpful ethical framework for genomic data that does justice to genomics’ growing expansion in society as well as its growing complexity. It helps to articulate and address a broad spectrum of ethical, social, and political factors in a variety of different societal contexts, while giving consideration to the interests of individuals, groups, and society at large.

## Limits of informed consent for genomic data in different societal contexts – lessons from online big data

Autonomy is a central concept in most bioethics discussions. It is often implied that autonomy is realized through individual consent. Yet, the predominant focus on individual autonomy and informed consent has been criticized, both in the bioethical and legal literature (Herring 2019; Lõhmus 2015; Nedelsky 2011; Rommetveit 2016; Stoljar 2011; ten Have 2001; Widdows 2013). The shortcomings of this focus have been discussed from various perspectives within the bioethical debate, and mostly focus on the medical (i.e. research and/or clinical) context. For instance, critics have pointed to the risk that consent might rest on a limited understanding of the information (Manson and O’Neill 2007), that patients may not receive the right information to make a decision, that information may be too complex and extensive to be sufficiently understood by patients^1^ (Alblas et al. 2019), that they could be coerced into making a decision, that many different other people can influence an individual decision (O’Neill 2001) that patients have biases and limitations in their reasoning (Kirchhoffer and Richards 2019; Levy 2014; Rommetveit 2016), or that current informed consent approaches often fail to account for factors such as oppressive socialization (Sisti and Stramondo 2015). Also, there is debate whether a liberal approach to autonomy and informed consent (Beauchamp 2011; Pugh 2020) should be replaced with a more relational approach to autonomy that takes into consideration that individuals are social, relational beings (Mackenzie and Stoljar 2000; Stoljar 2011). Furthermore, within the light of increasing amounts of data and novel technologies that give rise to new possibilities for medical research, a variety of different types of consent has been proposed for medical research, including blanket consent, broad consent, and open consent (McGuire and Beskow 2010; Rothstein et al. 2016).

These critiques, however, do not fundamentally question the centrality of informed consent itself. Rather, they are mostly critiques *within* the realm of consent (such as practical challenges to obtain valid consent or conceptual debates on what consent is or ought to be). This is not to say that these critiques are not important, but rather they raise the question whether we need to look further than consent when it comes to genomic data, *in particular* when it comes to societal contexts beyond the clinical setting. Because – even if we assume for the sake of the argument that some sort of utopian ideal consent has been given by an individual – there are *still* many situations in which informed consent should *not* be given a central role as an ethical justification for a certain practice.^2^ As Widdows has pointed out, an inadequate reaction is often “to force a wholly inappropriate one-size-fits-all ethical model onto all situations” (Widdows 2013). Instead of addressing issues and solutions to the limitations of informed consent *within* the realm of consent, what we might need is a radically new way of approaching ethical issues that arise from genomic information across different social contexts.

There are at least two elements that – taken together – may fundamentally challenge the centrality of the informed consent approach in genomic information in specific contexts. Namely, the informed consent approach does not do justice to (1) the *interconnected* nature of genomic information, and (2) the social *context* in which genomic information plays a role, resulting often in a too individualistic focus. I will discuss both elements. Furthermore, I show that these two elements can also be recognized in debates on the ethical issues of online Big Data practices.

Firstly, genomic information is often *shared*. Therefore, a decision of *one* person can affect many *others* that share part of their genomic data with that individual. The interconnected nature of genomic information can be compared with certain online Big Data activities. Here, too, a decision by one individual can affect (many) others. For example, in the Cambridge Analytica controversy, 270.000 Facebook users gave consent for their Facebook data to be used, but subsequently also the data of all their Facebook friends was collected, so eventually data was obtained of 87 million Facebook users (De Groot 2023b; Hu 2020; Véliz 2021) This points to what Nissenbaum has named the ‘tyranny of the minority’: the willingness of a few people to share private information can also disclose information about many others (Barocas and Nissenbaum 2014). Another example is the case of a company who had *inferred* a general rule of the relationship between certain shopping habits and pregnancy on the basis of a small proportion of women who had disclosed information about their pregnancy and birth to the company (Barocas and Nissenbaum 2014). In that way, they could recognize the signs in *other* (non-consenting) women.

This is even more relevant for genetic information. Here, too, information disclosed by one person can also disclose information about others. This can happen in a more or less ‘direct’ way, for example if one individual does a genetic test for a condition that involves a 50% chance their siblings are also affected. Or when someone is arrested for a crime because their third-degree cousin decided to make his commercial DNA test results accessible to law enforcement searches. But it could also happen in more indirect ways, such as when a member of an Indigenous population gives consent to participate in research, thereby also disclosing broader information about e.g. the Indigenous populations itself. It has been argued that group harms cannot be mitigated only by securing individual consent, because this does not take into account the possible harms to communities (Friesen et al. 2017). In other words, genetic and genomic information reaches beyond the individual to relatives and communities (Burgess 2001; Widdows 2013). This is also increasingly recognized in the legal context. For example, it has been argued, drawing on case law of the European Court of Human Rights, there is increasing judicial recognition “(…) that the enduring and diffuse nature of DNA data makes it particularly problematic from a privacy perspective, and implicates the interests of a *broader biological group* whose privacy is impacted when DNA is retained and processed.[emphasis added]” (Costello 2022; p. 16).

This shared nature of genetic information and the implications for broader groups challenge the centrality of individual consent. Informed consent can be an ethical panacea^3^ in some cases and an ethical conundrum in others. This is largely dependent on the specific *context.* This brings us to a significant concern: the medical conception of informed consent is extrapolated to *other* contexts in society, in which the overfocus on consent will not be able to bring into the light issues that arise *beyond* consent. In other words, this consent emphasis is not sufficiently sensitive to particular societal contexts. Here, too, an important analogy can be made with the online Big Data context. Nissenbaum argues that the predominant approach to protect online privacy is to focus on protecting consumers and protecting commercial information, thereby too narrowly addressing only the commercial domain (Nissenbaum 2011). According to her, conceptions of privacy that only focus on this particular context are inadequate (Nissenbaum 2011). Instead, we have to take into consideration the ‘radical heterogeneity of online activity and practice’ (Nissenbaum 2011).

The same is true for genomic information. Consent may be too individualistic in some cases and approaching consent “as an absolute moral principle result in a reductionist abstraction and an *empty ethics* that strips the principle of consent away from its social context.” (Corrigan 2003; p. 787). Furthermore, it does not do justice to the ‘radical heterogeneity’ of all possible societal contexts that genomic information is involved in. An over-emphasis on consent may not recognize broader ethical and political issues on the level of communities and groups, such as when it comes to genetic surveillance of minority groups. To what extent the interests of groups and communities are affected by individual decisions will often depend on the context.

There is an extensive debate on genomics and consent in the literature that addresses the challenges that consent faces and suggests novel types of consent (Chapman et al. 2023; Chow-White et al. 2015; Fisher and Layman 2018; Horton and Lucassen 2019). These alternative approaches to consent may well be able to address important issues of consent, but at the same time they often operate *within* the realm of consent, aiming to revise consent rather than replace it (Chow-White et al. 2015). While these alternative approaches are very helpful, especially for the medical setting, this paper explores a different route, that aims to be applicable across many different societal contexts. As genomic information expands exponentially through society, we need a framework that is capable of articulating and addressing ethical issues in a wide variety of social contexts, not limited to healthcare. One that takes into account that genomic data is often shared *and* increasingly integrated in many contexts of social life, just as online Big Data information is. The contextual integrity framework is such a framework. I will discuss this framework in the next section and explore its advantages for genomic and genetic information.

## The contextual integrity framework by Nissenbaum

The contextual integrity framework is a way to explore whether a new socio-technical system or practice is politically and ethically legitimate (Nissenbaum 2009). The contextual integrity framework has been developed in the context of Big Data and contemporary information technology systems in which complete individual control of information seems not feasible anymore. Nissenbaum argues that what people find important is not so much that they as individuals have complete *control* over information or that they want that no information is shared about them at all, but rather they think it is important that information is shared *appropriately* (Nissenbaum 2009). The sharing of information has to adhere to certain legitimate expectations on how that information *should* flow in a certain *context*: the context-relative informational norms. Every context (e.g. the healthcare context, the workplace context) has different norms that depend on the particular context. This is already visible in day-to-day life: one shares different information with one’s colleague at work than with one’s physician, because the values people find important (as well as which information people are willing to share) vary across contexts. Context-relative informational norms are not static, but can change through time, both quickly and slowly (Barocas and Nissenbaum 2014). Also, they can be implicit (such as social norms) or explicit (such as those embedded in legal systems) (Nissenbaum 2009).

The contextual integrity approach takes at its foundation that different norms exist for different contexts on *how* information should be shared. Contextual integrity is violated when those context-relative informational norms are breached. Public protest or resistance against novel technological information systems can often be traced back to breaches of contextual integrity (Nissenbaum 2009). The context-relative informational norms are constituted by the following four parameters: contexts, actors, attributes, and transmission principles (Nissenbaum 2009).

With respect to *contexts*, Nissenbaum holds that the often mentioned public-private dichotomy regards only *two* contexts out of many other social contexts, including the healthcare context, the workplace context, or the family context. *Actors* are distinguished in three types: information senders, recipients, and subjects (the people that the data is about). For example, in a healthcare context, patients seeking medical attention are the information senders and subjects and the physicians are recipients (Nissenbaum 2009). But information can be shared with others, for example physicians (senders) can share information with colleagues or medical students (recipients). Thirdly, *attributes* are information types, such as addresses or patients’ medical conditions. Context-relative informational norms regard asking about certain types of information (attributes) as appropriate or inappropriate, depending on the context. For instance, a physician asking you about certain mental or physical conditions is considered appropriate, but your boss making the same inquiries is usually considered inappropriate (Nissenbaum 2009). Lastly, *transmission principles* express terms and conditions to transmitting of certain data. A transmission principle can be e.g. confidentiality, reciprocity, consent, or compulsion, depending on the context. Transmission principles are, thus, constraints on information flows from one party to another and express the conditions under which information should or should not flow.

The central element of this framework is to evaluate a certain novel technology by checking whether this technology alters information flows in the different parameter fields (Nissenbaum and Patterson 2016). According to the contextual integrity framework, alterations in the information flows that are brought about by a new technology, can result in a privacy violation. For example, when employers are given access to health self-tracking apps, the parameter of *actors* changes in the sense that employers are added to the recipients (Nissenbaum and Patterson 2016). This is considered inappropriate because, in short, lifestyle information from the tracking app, originally intended only for the person herself, or maybe physicians, friends, or family, is now extended towards employers. In other words, context-dependent informational norms are breached. An important advantage of the contextual integrity approach is that it offers an alternative to accounts that merely focus on *one* factor, such as whether the data is ‘sensitive’ or not or a focus on consent as a justifiable reason to share data (Nissenbaum 2019). Instead, the contextual integrity framework focuses on all five parameters at once (Nissenbaum 2019). An example to illustrate this is the case of the Cambridge Analytica scandal. In this case, public outrage did not occur *only* a result of circumventing the consent of the subjects, but mainly focused on the unacceptability of Cambridge Analytica being a data recipient of Facebook data (Nissenbaum 2019).

When a violation of contextual integrity norms has been identified, the next step is to evaluate whether this practice is justified or not. This depends on the interests, goals, and ethical and political values of a certain context (Nissenbaum 2019). This evaluation is always needed, because banning a practice that breaches context-informational norms from the outset, would make the contextual integrity framework unnecessarily conservative (Nissenbaum 2019). Rather, the contextual integrity framework aims to respond to rapidly evolving technological developments, including those that raise new novel data flows (Nissenbaum 2019). This is also an advantage for genomic data, because many new genomic data flows may be beneficial: the contextual integrity framework will not create extra barriers in these instances, in which the flow of data aligns with the values and goals of the particular societal context in which they are used. The same applies for the other side: if, for example, a party proposes to share patient healthcare data with advertisers and prospective employers, the contextual integrity approach will articulate that the goals and values of the healthcare context are not upheld by this new practice (Nissenbaum 2019). Importantly, although contextual integrity was originally focused on privacy, it can also be used as a more broader conceptual tool that “can aid our thinking about how technological changes affect the full range of human values and concerns – not only privacy” (O’Neill 2022). The contextual integrity approach aims to broaden reflection and evaluation of ethical *and* political factors.^4^

When it comes to genomic information, I argue, contextual integrity serves as an effective ethical framework that is able to articulate and address ethical challenges that arise from genetics and genomics across a variety of societal contexts and does justice to the shared nature of genomic information as having implications for groups and communities. Moreover, the contextual integrity framework for genomic information flows enables us to articulate whether informed consent is relevant in a specific case. Informed consent is in the contextual integrity framework just one of many possible transmission principles. In turn, transmission principles are only *one* of the parameters to evaluate whether the context-relative informational norms are respected.^5^ Thus, the appropriateness of informed consent is dependent on the context, the types of information, the information subjects, the information sender, and the recipient. In other words, the contextual integrity approach does not refute the value of informed consent in general: rather, consent is considered to be just one of many possibilities to regulate the flow of data. For example, in the clinical-medical context of a doctor-patient relationship to share genetic data, the context-dependent information norms will often correspond to the consent-model. Contextual integrity looks further than the *practical* difficulties of informed consent and instead explores its more fundamental irrelevance (or, importantly, relevance) in certain cases involving genomic information.

In the next section, I will discuss several important advantages of the contextual integrity framework for genomic data.

## Advantages of contextual integrity for genomic information

In this section, I will discuss various advantages of the contextual integrity approach for genomic information. In so doing, I will explore multiple cases across several social contexts (research, clinical, forensic, commercial contexts etc.). The discussion of these cases will demonstrate how the contextual integrity framework, through a systematic examination of specific genomic information flows, helps to both articulate ethical challenges and guide ethical evaluation. When discussing contextual integrity’s advantages, I will also explicate in more detail why an over-emphasis on individual informed consent has limitations in multiple societal contexts.

### Sensitive *within* the medical context

The first major advantage of the contextual integrity framework for genetics and genomics, is that, while the contextual integrity approach is useful for a wide range of contexts, this by no means diminishes contextual integrity’s value for the *healthcare* context. On the contrary, the contextual integrity approach provides a useful tool for identifying and addressing ethical issues that arise from genomic information in the healthcare context, because of two reasons. First, the contextual integrity framework is able to effectively incorporate important ethical justifications and values that have been developed in the healthcare context. In other words, rather than suggesting a whole ‘new’ framework for articulating and addressing ethical challenges, it takes at its starting point values that are already ‘common place’ within bioethics (Nissenbaum calls these ‘entrenched norms’) in order to give guidance in *novel* situations that arise within genetics. In other words, in many ‘straight-forward’ clinical settings, the contextual integrity approach will not change current practice, while at the same time contextual integrity can serve as a useful tool to evaluate novel technologies or practices in genomics. Second, contextual integrity is sensitive to different situations *within* healthcare.

To illustrate these points, we can examine different cases within the medical contexts. Consider a case of a patient’s genetic testing results for Klinefelter Syndrome (a genetic condition characterized by an additional X chromosome of which symptoms can include learning problems, small testes size, and taller height). When it comes to the sharing of this information (the attribute of this information being the additional X chromosome which constitutes Klinefelter Syndrome) by, for example, the patient’s doctor, there are clear transmission principles that have been ‘entrenched’ for some time: most prominently, confidentiality and informed consent. As Klinefelter Syndrome is a non-hereditary genetic condition, the information is not shared with anyone else: this data is only about one individual i.e. one data subject. By contrast, the transmission principles and data subjects may vary from cases in which the genetic condition *is* hereditary, e.g. with genetic testing for a BRCA mutation. If a patient has a BRCA1 mutation, she has a 60–80% chance to get breast cancer during her lifetime and a 30–40% chance for ovarian cancer. The siblings and children of a patient with a BRCA1 mutation have themselves a 50% chance of also carrying this mutation. This means there are more *data subjects* than in the Klinefelter case: the patient with Klinefelter is the only data subject whereas in the BRCA1 case the *relatives* of the patient are also data subjects. This also has implications for the transmission principles. For cases like BRCA, the ‘joint account model’ has been developed, which holds that results of clinical genetic tests belong to the family and are therefore shared by the doctor with family members, like a bank manager sharing information of a joint bank account with all account holders (Parker and Lucassen 2004). In other words, when a doctor shares information with, for example, a sister of a patient, it would violate contextual integrity in the Klinefelter case but would not in the BRCA case. Therefore, contextual integrity is able to explore in a relatively quick and clear way how these cases are different from one another. Clearly, the contextual integrity framework is not the *only* way to achieve this, but it can still be valuable because it is broadly applicable: from more ‘easily’ distinguishable cases to more complex ones (and it will not always be clear from the outset which cases will turn out to be complex). Furthermore, contextual integrity will not unnecessarily stir up carefully established best practices in the clinical setting.

Another medical-related context is biobank research. There is an enormous variety when it comes to genetic biobank research, such as a variety in (future) risks or benefits for individuals and/or society, the number of research participants, the number of genetic relatives that is in some way affected by the research, and the type of research. This diversity entails that ethical issues and reflection vary greatly depending on many factors. Informed consent is often an important transmission principle in biobank research: only if informed consent from an individual patient has been obtained, information can be shared. Other proposed principles of biobank research are solidarity and benefit sharing (Chadwick and Berg 2001). Solidarity recognizes participants’ willingness to contribute to public medical research and counters exclusively autonomy-based governance of research biobanks (Prainsack and Buyx 2013). Individual consent, broad consent, and solidarity are only a few examples of the many different transmission principles that have been proposed for biobanks and I will not discuss them here in detail. What is important, is that they are tailored for a specific *context* within biobank research. For instance, in biobank research on frequently occurring genetic diseases, sharing information based on solidarity might be justified, but this may not be the case when it entails a mental disease in a certain minority group, as e.g. the risk of stigmatization is also a relevant factor. Therefore, ‘one size fits all’ approaches to biobanks, like the GDPR, are arguably not able to provide effective guidance within biobank research (Indrakusuma et al. 2020). Contextual integrity offers precisely the opposite of such a one-size-fits-all model. It allows for different context-relative informational norms for different types of biobank research (Indrakusuma et al. 2020). This is an important contribution of the contextual integrity approach, as it has been argued that effective governance of genomic research projects has to be sensitive to contextual factors (O’Doherty et al. 2021).

However, in the above cases of clinical genetic testing and biobanks, the extent to which contextual integrity can substantially contribute is arguably limited, as there may often already be clear and agreed-upon rules on how to handle these data. Indeed, the contextual integrity framework’s strength is probably most clearly visible in more complex cases as well as in future uses of genomic data. These future applications and implications of genomic data are still uncertain and difficult to foresee on the moment that e.g. genomic data is donated for research, so that consent is often broadly and open-endedly formulated and can be changed through time (Horton and Lucassen 2023). In that light, it has been argued: “The limits of consent place a huge responsibility on people holding genomic data to justify any action they might take with these data – rather than acting as a *carte blanche*, the necessity of asking consent in broad terms increases the need to consider very carefully how to work with genomic data *in a way that respects the reasons for which it was contributed.*[emphasis added]” (Horton and Lucassen 2023; p. 41). This notion of respecting the reasons is precisely what the contextual integrity framework has in mind: it should not only be about individual control over the data, as this may be (even with adapted versions of informed consent, such as broad consent) often infeasible, but also about the values, reasons, ends, and interests of a particular context of genomic research. Considering the variety of different applications within clinical genomics as well as in genomic research, let alone in contexts *outside* of the clinical-research setting, there will be an accordingly variety of contextual integrity norms and the values and ends to uphold, while they will plausibly also have common denominators, such as trust. To illustrate, asking people from underrepresented groups to participate in genomic datasets can be important to ensure more equity, but can also raise questions such as the risk of exploitation (Horton and Lucassen 2023).

The contextual integrity framework may aid our thinking in this quickly changing and expanding field of genomic research and clinical genomic testing. With respect to the sharing of genomic datasets, privacy concerns include the prediction of observable features, identification, and phenotype inference (Bonomi et al. 2020).There is a rapid growth of genomic data and its applications, such as genome-wide association studies, direct-to-consumer genomic testing, and the possibility of capturing human genomic data from environmental samples (Uffelmann et al. 2021; Whitmore et al. 2023). All these different types and uses of genomic data give rise to varying ethical issues. One significant overarching ethical issue is the potential implications for groups and communities (Chapman et al. 2023; Uffelmann et al. 2021), that vary depending on the context. In the next section, the advantage of contextual integrity’s sensitivity to group interests will be discussed.

### Sensitive to Group Interests

Another significant advantage of the genomics-specific contextual integrity approach, is that the framework is sensitive to group interests. This can be illustrated by discussing the case of genomic studies of Indigenous populations. Tsosie et al. (2019) argue that “(g)enomic studies often rely on individual-based consent approaches for tribal members residing outside of their communities. This consent model fails to acknowledge the risks to small groups, such as Indigenous populations, which can implicate the community as a whole”. Especially with small groups, such as tribal communities, the likelihood is high that one individual’s genomic data can be used to make inferences about the entire community. Tsosie et al. discuss the common practice that Indigenous individuals are recruited for genetic research through ‘overprioritizing’ individual informed consent models, thereby circumventing consultation of the population itself:

“This practice of recruiting Indigenous individuals from urban centres is not new; state epidemiological databases (…), hospital-based studies and universities partnered with direct-to-consumer genetic testing companies have long utilized and overprioritized individual consent models to passively or actively recruit populations that include Indigenous persons without tribal consultation.” (Tsosie et al. 2019).

This case shows what often goes wrong. It is not so much individual informed consent *itself* that is problematic, but rather that the individual informed consent model is applied to a particular *context* where this consent framework has significant limitations: it does not do justice to the interests of the group. Or, to use the phrasing of Widdows, a common practice is “to begin with the ethical framework and then to try to make the situation fit. This is the wrong way around.” (Widdows 2013; p. 167). This is where the contextual integrity approach shows its strength. The contextual integrity approach does not depart from the consent model, but departs from exploring this particular situation within a particular context. In this case, the contextual integrity approach would identify the community-based participatory research models in line with Indigenous communitarian ethics (Tsosie et al. 2019) as the appropriate transmission principle and the relevant data *subjects* to also include specific groups.^6^ When one starts with this context evaluation, one can effectively identify the context-relative informational norms thereby circumventing the major risk of inadequately imposing a consent model on the wrong context. Tsosie et al. (2019) have argued there is a “fundamental lack of acknowledgment of risk to groups in standard informed consent procedures”. Furthermore, contextual integrity allows a more fine-grained evaluation when it comes to different types of genetic biobank research. For example, in a biobank such as UK Biobank, the interests of biobank participants could be more regarded as an accumulation of individual interests, and a clear group interest, such as in the case of genetic research of Indigenous populations, is less relevant here. Widdows has argued that with UK Biobank, “their group right does reduce to their individual interests and they are a group in no other sense than in their shared interests of being treated fairly by UK Biobank” (Widdows 2009). The normative implication of that is contextual integrity will, in some cases, pinpoint informed consent as a central element, because the interest of this group can be reduced to the individual interests. Therefore, individual-based concepts such as informed consent are within this particular context an important parameter of the context-relative norms of how to share genetic data within UK biobank. However, depending on the type of biobank research, there could also be interests on the level of a group qua group, such as in the case of Indigenous populations.^7^ A contextual integrity approach allows for tailored consideration for differences in types of groups, in cases where an informed consent model risks handling all genetic biobank research as the same. By contrast, whether contextual integrity is respected or violated, depends (among other parameters) on the type of group that is involved in a particular case. In sum, the contextual integrity approach, can do justice to group interests in cases where the individual informed consent model has significant constraints.

The contextual integrity framework can also contribute to identify and articulate ethical issues when it comes to genetic research of other vulnerable populations. For example, it has been argued that while non-governmental groups and legal scholars are advocating for genetic rights for Indigenous populations in the US and Australia, this is often not the case for other vulnerable populations that are considered to be foreign in their countries, such as the Roma people in Europe, Tibetan people in China, or Kurdish communities in Turkey (Lipphardt et al. 2021). In other words, it might be that no formal guidelines do exist as they do for Indigenous populations in Australia. Still, these groups’ interests will *also* have to be protected. A contextual integrity approach would be able to identify the group interests of these populations. For transmission principles on how this genetic/genomic data may be shared in these contexts, one could take as a departure point the CARE principles for Indigenous Data Governance (Carroll et al. 2021; Lipphardt et al. 2021) to also be used for these populations.

Furthermore, novel developments in genomics can reinforce potential group-based harms (Chapman et al. 2023; Leib-Neri and Prince 2022). These may align with groups that are socially regarded as a group, such as particular ethnic groups. For example, genomic research could raise concerns about reputational harm and reveal sensitive information about a group’s history (de Vries et al. 2020). But genomics could also establish groups based on untransparent algorithmic analysis of genomic data, whose members have no idea they have been categorized as being part of this group (Hallinan and De Hert 2017). Artificial intelligence or machine learning analysis of genomic data may accelerate the creation of these groups and may lead to consequences that far extend beyond the initial research (Chapman et al. 2023). An example of this could be capturing human genomic data from environmental samples, which may also result in group harms (De Groot 2023a).

In sum, contextual integrity reveals why informed consent in UK Biobank is an important element on the basis of how genetic information can be shared, but arrives at a different conclusion with specific genetic studies to Indigenous populations or different types of genomic research. Contextual integrity is sensitive to the broad variety of different types of genetic/genomic research, that can be explored by the different contexts, data subjects (including groups), information types, and information principles.

### Sensitive across different societal contexts and moving beyond consent

Another advantage of the contextual integrity framework for genomic information, is that its usefulness is not limited to the medical context, but is broadly applicable across a wide variety of contexts, such as genetic surveillance or commercial genetic testing. With an individualist focus on consent there is the risk that significant ethical, social, and political concerns will not be brought into the light, as this section will illustrate. Contextual integrity allows for consideration of elements beyond individual control and beyond the medical contexts.

Importantly, the contextual integrity framework can aid our thinking about an increasingly frequent phenomenon, namely that genomic information often *crosses* different contexts. Examples are forensic use of medical biobanks, research use of commercial genomic data, forensic use of commercial genomic data, or insurance use and employer use of medical genetic data. Contextual integrity can offer clear guidance in these cases, as it starts with the particular *context*. For example, with forensic use of medical biobanks, the contextual integrity approach would reveal that information flows are altered in several parameter fields. Namely, law enforcement would become an additional information recipient. Also, the transmission principles in medical biobanks, e.g. trust and confidentiality (more specifically that people rely their data will be only used for research purposes or, when not, with their consent) would change. In the evaluation of this breach of contextual informational norms, one will have to explore whether the goals, ends, and values of the healthcare context are threatened. This seems to be the case: because the contextual informational norms are breached if law enforcement gets access to this data, people may refrain from seeking medical help, may risk their health and possibly the health of others, creating problems for the accessibility of healthcare and negatively impact doctor-patient confidentiality and trust in the medical profession (De Groot, Van Beers, Decock, et al., 2021). These are all important goals and values of the context of the medical clinical setting. Therefore, the practice of giving law enforcement access to medical genetic databases is, it can be argued, not in line with the values, goals, and interests of this medical context. If we accept that conclusion, then, according to the contextual integrity framework, such use lacks legitimacy. An important advantage of the contextual integrity framework in this case is that it considers all five parameters that constitute context-dependent informational norms and also takes into account which values and ends are central and which are more peripheral. To illustrate, if we think of a case of police getting access to the medical genetic data of a patient admitted in a hospital to check their DNA profile with DNA from a crime scene, we may reason from a consent-perspective that this is wrong because the patient has not given consent. But the contextual integrity framework is able to articulate the broader social issues that are important within this context and to reflect on the social and ethical implications for the medical clinical context as a whole.

Similarly, employer use of medical genomic data will also result in altered information flows. Another example could be pharmaceutical drug production use of commercial 23andMe genetic data of DNA test consumers (Van Dijck and Poell 2016). For example, in 2018, 23andMe sold access to the data of around 5 million people to the pharmaceutical company GlaxoSmithKline for 300 million dollar (Fox 2020).^8^ Earlier, 23andMe had sold another pharmaceutical company access to its database of DNA profiles of people with Parkinson’s disease for 60 million dollar (Van Dijck and Poell 2016). This is another case in which contextual integrity can be helpful to articulate and address complex and broad issues. While the rhetoric of 23andMe focuses on this data’s use for the ‘public interest’ of doing useful research, raw genetic data is commodified and privatized (Van Dijck and Poell 2016). Contextual integrity, by considering multiple parameters and focusing on the goals and values within a particular context, can help to explore the negative effects when public good and corporate gain are deliberately and non-transparently intertwined by these commercial actors. Thus, when genomic information sharing *crosses* contexts, contextual integrity would be an accessible way to articulate what changes precisely in these information flows.^9^ This is, importantly, not to say that it would *always* be morally impermissible. For example, there might very well be cases in which a novel practice is in fact *better* in protecting the ethical, political, and context-based values of a particular context, such as certain novel encrypting methods of genomic information. Another example could be the case of COVID-19-related genomic data sharing (Maxwell et al. 2023): the evaluation may very well identify that a certain novel practice contributes beneficially in upholding the goals and values of the public health setting.

Contextual integrity can also identify significant ethical problems, such as in the case of investigative genetic genealogy (De Groot 2023b). With investigative genetic genealogy, a DNA profile from a crime-scene is uploaded to a commercial DNA database, hoping to find a distant relative of the suspect. Through this relative, an expert can examine the intersections of the family trees of this relative and the unknown suspect, narrowing the group of suspects down to one person or a number of siblings. When law enforcement searches commercial DNA-databases, all five parameters that constitute contextual-dependent informational norms are altered (De Groot 2023b). More important, however, is the fact that a focus on individual consent will risk creating a ‘tyranny of the minority’ in this case (Barocas and Nissenbaum 2014; De Groot 2023b), something contextual integrity will prevent. When focusing on consent, it has been estimated that only 2% of a population needs to consent to have their DNA profile to be included in a commercial DNA database open for law enforcement to be able to track down almost anyone in that population through IGG (Erlich et al., 2018). This is because the DNA profiles used with commercial genetic testing are very extensive and look at hundreds of thousands different parts of the DNA. In other words, the individual consent of one individual has consequences for thousands of relatives. When 2% of the population decides to participate in police access, they make indirectly a decision for the entire population: a tyranny of the minority (De Groot 2023b). An over-emphasis on individual consent leaves some significant ethical and social-political concerns unaddressed.

By contrast, contextual integrity takes a broader viewpoint and also considers political factors that are affected by this practice. It looks at the effects on “power structures, implications for justice, fairness, equality, social hierarchy, democracy, and so on” (Nissenbaum 2009; p. 182). When one examines the public debate on investigative genetic genealogy, one sees it quickly falls back on questions of the ‘practical issues’ of consent. These are questions such as whether DNA test consumers have really consented to police usage of their data, if they have perhaps not changed their mind after consent, or whether they had to actively click or actively unclick the box that says they consent to police use of their data (De Groot 2023b). However, the ‘tyranny of the minority’ makes these questions almost irrelevant. Namely, even if we assume that some ‘ideal’ consent has been given, there *still* remains the problem of the tyranny of the minority (De Groot 2023b). Contextual integrity enables changing the course of the debate from the myopic focus on the practical challenges of consent to considering the full range of ethical and social-political issues, including the importance of democratic decision-making on this issue (instead of leaving it to the tyranny of the minority), the involvement of commercial parties, and the implications for the relation between state and citizens (De Groot 2023b).

Thus, contextual integrity is a valuable tool to look beyond individual control (and, thus, informed consent) over data and beyond the medical contexts, thereby avoiding treating genetic information as if it always exists in a non-political vacuum.

## Discussion

As we have seen, an over-emphasis on individual consent and individual control over data risks pushing important ethical issues out of sight. Importantly, this paper does not suggest to disregard consent entirely. Yet, it is important to explore when informed consent should be the main basis of the ethical debate *and* when it should not. The bioethical debate on genomic and genetic information needs to put more emphasis on the *shared* nature of genetic data as well as the fact that genetic information expands through many capillaries of society and, therefore, our social life.^10^ This paper presented the contextual integrity framework as a theory to do justice both to this shared nature as well as the different societal contexts that genetic data plays a role in. In the online privacy and Big Data debates, the theory of contextual integrity has aided over the past years to move the debate beyond individual privacy and individual data control. For genetic data, it can also contribute in similar ways to moving the bioethical debate beyond the individual. The contextual integrity framework is able to articulate ethical *and* political issues that are involved with certain practices, and is context-sensitive, also within the healthcare context. These insights can help to identify effective ways of governance.

A benefit of the contextual integrity approach is that it probably appeals to both proponents of a relational conception of autonomy as well as to proponents of a more liberal, individual conception of autonomy. It has been argued that *independence* of individuals controlling their information is in this age obsolete: rather, an individual is *interdependent* (Lõhmus 2015) and the self is a ‘relational self’ (Herring 2019; Nedelsky 2011). For Widdows, the self is the ‘connected self’ (Widdows 2007). In the case of genetic information, the self is – even when one holds a strictly individual conceptualization of the self as being independent and rational in making *decisions* – still attached to others in a very literal sense: the self is a ‘shared self’. The ‘self’ inevitably shares parts of their genetic information with others. Yet, to what extent the self is a shared self depends highly on the *context.* There are situations concerning genetic information in which the self can still be constructed as mainly an independent individual self (the world individual stems from the Latin *individua*: inseparable, indivisible), such as in the Klinefelter case. But in many other societal contexts, the fact that an individual shares parts of their genetic data with others has broader implications. To approach the self in *every* societal context as an individual self, instead of a shared self, creates the risk that not all relevant ethical and political issues come into the light. What remains, for example in the consent-focused debate on forensic use of commercial DNA databases, is a limited debate unable to look beyond individual interests of a small group of genetic testing consumer enthusiasts. It is almost as if the ethical debate on the desirability of self-driving cars would revolve primarily around the type of seatbelts that should be installed in these cars. Surely, seatbelts are a relevant consideration, but all would agree that this is not the *main* ethical question that has to be asked about self-driving cars.

Contextual integrity could also be a helpful tool for the debate on genetic exceptionalism. The genetic exceptionalism debate revolves around the question whether genetic data is really that different (‘exceptional’) from other sensitive data, such as health-related data. The contextual integrity approach may be helpful in this debate, because whether or not genetic data is exceptional, depends, I think, on the context. In the Klinefelter case, sharing genetic data showing the extra X chromosome or sharing a note from the patient’s medical files saying ‘patient is diagnosed with Klinefelter Syndrome’ do arguably not differ in any morally significant way. However, this is plausibly different when an entire commercial DNA profile looking at 700.000 different places in the genome is shared with a pharmaceutical company.

The bioethical debate on genetic information must accept that there is not a single top-down moral principle or approach (such as consent) that can be applied to *all* situations. Rather, the relevance of concepts, norms, and principles will almost always be context-dependent. The contextual integrity framework is a helpful tool to address these fine-grained differences.

## Conclusion

When genomic information expands further and further in society, far beyond the medical setting, we need bioethical reflection that equally looks beyond that setting. More specifically, as I have discussed, we need to look further than approaches based on individual informed consent and individual control, because although these are often useful for the medical setting, they have significant limitations in other societal contexts where genomic information plays an increasingly significant role. To confront these challenges, this paper suggested a contextual integrity framework, founded within Big Data ethics and modern information technology. It explored several advantages of this framework, including that it enables to articulate the irrelevance of informed consent in *specific* context-dependent situations. The contextual integrity framework contributes to moving the bioethical debate on genomic data beyond the medical setting and beyond individual consent. It helps to articulate and address a broad spectrum of ethical, social, and political factors in a variety of different societal contexts, while giving consideration to the interests of individuals, groups, and society at large.

## Footnotes

1 For example, the terms of services of direct-to-consumer genetic testing companies are currently often very complex and mimic ‘accept-all-cookies’ approaches. Often these companies make use of very long so-called ‘clickwrap contracts’ (Phillips 2019).

2 Meanwhile, clearly, alternatives to consent exist, also in the healthcare setting: in multiple cases, consent does not play a primary role, e.g., when it comes to report certain infectious diseases, or quarantine, considered vital to the public interest.

3 Various authors have emphasized the problem of regarding informed consent as an ‘ethical panacea’ (Corrigan 2003; Laurie 2002; Lyle et al. 2022; Samuel and Kennett 2020). Barocas and Nissenbaum have argued that ‘[W]hile the idea that informed consent *itself* may no longer be a match for challenges posed by big data (…), such thinking has not entered the mainstream [emphasis original]”(Barocas and Nissenbaum 2014).

4 Nissenbaum states “Consider moral and political factors affected by the practice in question. (…) What might be the effects on power structures, implications for justice, fairness, equality, social hierarchy, democracy, and so on?” (Nissenbaum 2009).

5 See Nissenbaum’s discussion on control (p. 148).

6 Although Nissenbaum takes data subjects to be single individuals and does not explicitly consider group interests or group privacy (Nissenbaum 2009; p. 253), the framework does allow for (many) other individuals to be considered data subjects.

7 Widdows, within the context of medical genetic data, adopts from the legal debate on group rights the distinction between *corporate* groups and *collective* groups (Jones 1999; Widdows 2013). Widdows’ approach is in line with Nissenbaum’s approach in the sense that it also critically looks at the predominance of informed consent and individual control models. Within a *collective* group, the interests of the group are always reducible to the sum (a ‘collection’) of individual interests (Widdows 2013). When we refer to the interests of such a group we are only referring to the sum of the individual group-members interests. In contrast, with a *corporate* group, the right of the group is *its* right: the interests of the group are *not* reducible to individual interests (Jones 1999; Widdows 2013).

8 For other applications of contextual integrity in the context of commercial genetic testing, see: (Huang and Bashir 2015)

9 With genomic data being involved in more and more societal contexts and the crossing of these contexts, future work could focus on the challenges to consensus on what the (changing) norms are in various contexts.

10 The cases that were discussed in this paper can by no means offer an exhaustive account of all the social contexts in which genomic information plays a role. Contextual integrity can be a helpful tool for multiple other practices that were not discussed in this paper.
